# Differential lung gene expression changes in C57BL/6 and DBA/2 mice carrying an identical functional *Mx1* gene reveals crucial differences in the host response

**DOI:** 10.1186/s12863-024-01203-3

**Published:** 2024-02-15

**Authors:** Silke Bergmann, Linda Brunotte, Klaus Schughart

**Affiliations:** 1 Department of Microbiology, Immunology and Biochemistry, University of Tennessee Health Science Center, Memphis, TN USA; 2 Institute of Virology Münster, University of Münster, Von-Esmarch-Straße 56, 48149 Münster, Germany

**Keywords:** Influenza, Mouse, C57BL/6J, DBA/2J, MX1, Transcriptome, Lung

## Abstract

**Background:**

Influenza virus infections represent a major global health problem. The dynamin-like GTPase MX1 is an interferon-dependent antiviral host protein that confers resistance to influenza virus infections. Infection models in mice are an important experimental system to understand the host response and susceptibility to developing severe disease following influenza infections. However, almost all laboratory mouse strains carry a non-functional *Mx1* gene whereas humans have a functional *MX1* gene. Most studies in mice have been performed with strains carrying a non-functional *Mx1* gene. It is therefore very important to investigate the host response in mouse strains with a functional *Mx1* gene.

**Results:**

Here, we analyzed the host response to influenza virus infections in two congenic mouse strains carrying the functional *Mx1* gene from the A2G strain. B6.A2G-*Mx1*^*r/r*^(B6-*Mx1*^*r/r*^) mice are highly resistant to influenza A virus (IAV) H1N1 infections. On the other hand, D2(B6).A2G-*Mx1*^*r/r*^(D2-*Mx1*^*r/r*^) mice, although carrying a functional *Mx1* gene, were highly susceptible, exhibited rapid weight loss, and died. We performed gene expression analysis using RNAseq from infected lungs at days 3 and 5 post-infection (p.i.) of both mouse strains to identify genes and pathways that were differentially expressed between the two mouse strains. The susceptible D2-*Mx1*^*r/r*^ mice showed a high viral replication already at day 3 p.i. and exhibited a much higher number of differentially expressed genes (DEGs) and many DEGs had elevated expression levels compared to B6-*Mx1*^*r/r*^ mice. On the other hand, some DEGs were specifically up-regulated only in B6-*Mx1*^*r/r*^ mice at day 3 p.i., many of which were related to host immune response functions.

**Conclusions:**

From these results, we conclude that at early times of infection, D2-*Mx1*^*r/r*^ mice showed a very high and rapid replication of the virus, which resulted in lung damage and a hyperinflammatory response leading to death. We hypothesize that the activation of certain immune response genes was missing and that others, especially *Mx1*, were expressed at a time in D2-*Mx1*^*r/r*^ mice when the virus had already massively spread in the lung and were thus not able anymore to protect them from severe disease. Our study represents an important addition to previously published studies in mouse models and contributes to a better understanding of the molecular pathways and genes that protect against severe influenza disease.

**Supplementary Information:**

The online version contains supplementary material available at 10.1186/s12863-024-01203-3.

## Background

 Influenza virus infections represent a major global health problem. Severe pandemics caused by zoonotic influenza A virus (IAV) strains that circulate in migratory and wild birds pose a concerning risk, as exemplified by the Spanish flu in 1918 that resulted in about 30 million deaths worldwide [1, 2]. In addition, human-adapted IAV of the H1N1 and H3N2 subtypes cause seasonal epidemic outbreaks with high mortality among elderly and immunocompromised individuals and large economic losses every year [3].

The MX dynamin-like GTPase 1 (MX1) gene is one of the most powerful host restriction factors against viral pathogens in mammals and is induced in response to the expression of type I and III interferons in infected cells and tissues [4–10]. The stalk domain of MX1 is able to form a ring-like oligomer that is thought to interact directly with intracellular viral ribonucleoproteins (vRNPs) to block viral RNA synthesis, including viral mRNAs and new genomic RNAs [4–10].

The mouse is an important model system to study the pathophysiology as well as the biological mechanisms and activation of gene regulatory pathways during influenza A infection and disease. However, in contrast to humans, most laboratory mouse strains carry a mutation in the *Mx1* gene, which renders it non-functional and limits its comparability to humans [4–10].

Therefore, it is important to study the host response to influenza A infections in mouse strains that carry a functional *Mx1* gene. Here, we utilized two strains, C57BL/6J (B6-*Mx1*^*r/r*^) and DBA/2J (D2-*Mx1*^*r/r*^), that carry the same functional *Mx1* gene. Both strains have been generated by back crossing to the A2G strain, making them congenic for the same functional A2G *Mx1* allele [11]. Both strains have already been shown to serve as important models for studying influenza A host response and disease in vivo in the context of a functional *Mx1* gene (e.g. [12, 13]. C57BL/6J is the most commonly used mouse strain for many biological studies. In addition, the majority of gene knockout studies have been performed on the C57BL/6J genetic background. However, its *Mx1* gene is non-functional. C57BL/6J mice carrying the functional *Mx1* gene of the A2G mouse strain (B6-*Mx1*^*r/r*^) were shown to be highly resistant to lethal A virus infections [8, 14–17]. On the other hand, the same functional *Mx1* gene in the DBA/2J mouse strain (D2-*Mx1*^*r/r*^) does not confer protection against severe disease after influenza infection [11]. D2-*Mx1*^*r/r*^ mice exhibit very high viral loads early after infection as well as a hyper-inflammatory response in the lung, which causes high levels of immune cell infiltration and damage to the lung [11]. Consequently, D2-*Mx1*^*r/r*^ mice rapidly lose body weight after infection and die [11]. Intriguingly, pretreatment of D2-*Mx1*^*r/r*^ mice with interferon [11] or with defective interfering particles protects D2-*Mx1*^*r/r*^ from severe IAV disease [12, 13] and death, most likely because functional MX1 protein is produced before infection, thus inhibiting the rapid spread of virus in the early infection phase. Humans carry a functional *MX1* gene but may still experience severe influenza disease. Therefore, D2-*Mx1*^*r/r*^ mice carrying a functional *Mx1* gene represent an improved in vivo model for investigating the host responses and possible intervention strategies for severe influenza infections and disease in humans.

Here, we performed a transcriptome analysis to study changes in gene expression in the lung after influenza A virus infection in mice with a functional *Mx1* gene. B6-*Mx1*^*r/r*^ were resistant and survived, D2-*Mx1*^*r/r*^ mice were highly susceptible and died. We identified many differentially expressed genes at days 3 and 5 post-infection (p.i.) versus mock-treated controls in both strains, and between the two strains. D2-*Mx1*^*r/r*^ mice exhibited a hyperinflammatory response, with many genes more strongly expressed than in B6-*Mx1*^*r/r*^ mice, yet some DEGs were specifically up-regulated only in the resistant B6-*Mx1*^*r/r*^ mice.

## Methods

### Aim and design of the study

The aim of the study was to identify differentially expressed genes after infection of B6-*Mx1*^*r/r*^ and D2-*Mx1*^*r/r*^ mice with influenza A virus compared to mock-treated controls and between the two strains. For this, female mice of both strains were infected with the PR8 influenza A virus, and RNA was isolated from lungs at days 3 and 5 post-infection (p.i.) as well as mock-infected controls and sequenced by next-generation RNA sequencing. Subsequently, levels of gene expression were compared between infected mice and controls and between the two strains and then analyzed by various bioinformatic methods. Five biological replicates (mice) were used per group.

### Viruses

PR8 (H1N1) Influenza A virus was propagated and titrated as described [18, 19]. Briefly, embryonated chicken eggs were incubated at 37 °C with 50–70% humidity and rotated regularly. On day 10, eggs were infected with dilutions of viruses (e.g., 10^−3^, 10^−4^). The blunt sides of the eggs were disinfected with iodine, a hole was pierced in the eggs, and 200 µl of virus solution was injected. Afterward, the injection side was sealed with glue. Eggs were incubated for 48 h at 37 °C and 50–70% humidity without rotation. After incubation, eggs were stored overnight at 4 °C. For virus harvest, the eggs were opened with a knife and the outer membranes were removed. The fluid was extracted with a pipette into a 15 ml tube and stored on ice. After the collection of the virus, an HA assay was performed to test for active virus. Tubes with active viruses were pooled, aliquoted and stored at -70 °C. Titer of the virus was determined by the focus-forming unit (FFU) assay, which is identical to the plaque-forming unit (PFU) assay, only plaques were identified by antibody staining. The methods have been described previously in detail [18].

### Mouse infections

Generation of mice on a C57BL/6 and DBA/2 background carrying a functional *Mx1* allele, B6.A2G-*Mx1*^*r/r*^ (B6-*Mx1*^*r/r*^) and D2(B6).A2G- *Mx1*^*r/r*^(D2-*Mx1*^*r/r*^) was described previously [11]. Both mouse strains were originally obtained from the Helmholtz Center of Infections Research, Braunschweig, Dept. of Infection Genetics. Consent for using these mouse strains was obtained by the owner (Klaus Schughart, then HZI). Experimental mice were bred and housed at the Laboratory Animal Care Unit (LACU, UTHSC Memphis). Ten to 12-week-old female mice were infected intranasally with 2 × 10^3^ FFU of PR8F virus in 20 µl PBS as described before [18, 20]. Mice were euthanized at the indicated days post infection (p.i.) with an overdose of isoflurane followed by cervical dislocation. Studies were performed with five mice per group.

### RNA preparation

Lungs were collected individually, washed in PBS and RNAlater and kept in RNA Later solution overnight at 4 °C and afterward at -70 °C. For RNA isolation, we used the RNeasy Midi kit from Qiagen. Lungs were thawed and transferred into lysing matrix D tubes containing 1 ml lysis buffer for RNA extraction. Then, individual whole lungs were homogenized by the FastPrep-24 Instrument (MP Biomedicals) for 2 × 1 min at 5G. Quality and integrity of total RNA were controlled on the 5200 Fragment Analyzer System (Agilent Technologies). RNA quality was confirmed on a 2100 Bioanalyzer Instrument (Agilent).

### RNAsequencing

The RNA sequencing library was generated from 500 ng total RNA using the Dynabeads® mRNA DIRECT™ Micro Purification Kit (Thermo Fisher) for mRNA purification, followed by the NEBNext Ultra™ II Directional RNA Library Prep Kit (New England BioLabs). The libraries were treated with Illumina Free Adapter Blocking and were sequenced on Illumina NovaSeq 6000 using the NovaSeq 6000 S1 Reagent Kit (300 cycles, paired end run 2 × 150 bp) with an average of 7 × 10^7^ reads per RNA sample.

### Bioinformatic analysis of RNAseq data

Reads were quality checked with package FastQC (version 0.11.4, http://www.bioinformatics.babraham.ac.uk/projects/fastqc), then trimmed using Trimgalore (version 0.4.4, [21]) with default settings. Trimmed reads were mapped to mouse genome annotation mm11 (ENSMBL Musmusculus.GRCm39 release 104) and the eight virus genome segments of PR8 virus [19] using STAR (version 2.5.2b, [22]) with default settings. Mapped mouse reads were counted using RsubRead (version 1.32.4, [23]). Raw counts of mouse mapped reads were then normalized and log_2_ transformed using the function rlogTransformation(dds, blind = TRUE) from the DESeq2 package (version 1.16.1, [24]) and an increment was added to the normalized values to make all values positive. Raw counts of virus mapped reads were normalized as counts per million reads (CPM). Principal component analysis (PCA) was then used to visualize variation among and between treatment groups. For the identification of differentially expressed genes (DEGs), the DESeq2 package (version 1.16.1, [24]) with the model DESeqDataSetFromMatrix(countData = countData, colData = coldata, design = ~ group) was used. All six treatment groups, infected B6-*Mx1*^*r/r*^and D2-*Mx1*^*r/r*^ mice at days 3 and 5 p.i., and mock-treated B6-*Mx1*^*r/r*^and D2-*Mx1*^*r/r*^ controls were included in the model. DEGs were determined by contrasting the groups from the model, based on an adjusted *p*-value of < 0.05 and exhibiting more than a 2-fold (log_2_ = 1) difference in expression levels. Multiple testing adjusted *p*-value were calculated according to Benjamini and Hochberg [25]. Functional analyses of DEGs were performed using the R software package clusterProfiler (version v3.14.3; [26]). Further analysis and visualization of expression data were performed using the R software package [27]. Hallmark gene sets [28] were downloaded from [29] and the mouse mh.all.v2022.1.Mm.symbols.gmt gene list was used for analysis.

## Results

### Body weight loss and viral gene expression

**Fig. 1:**
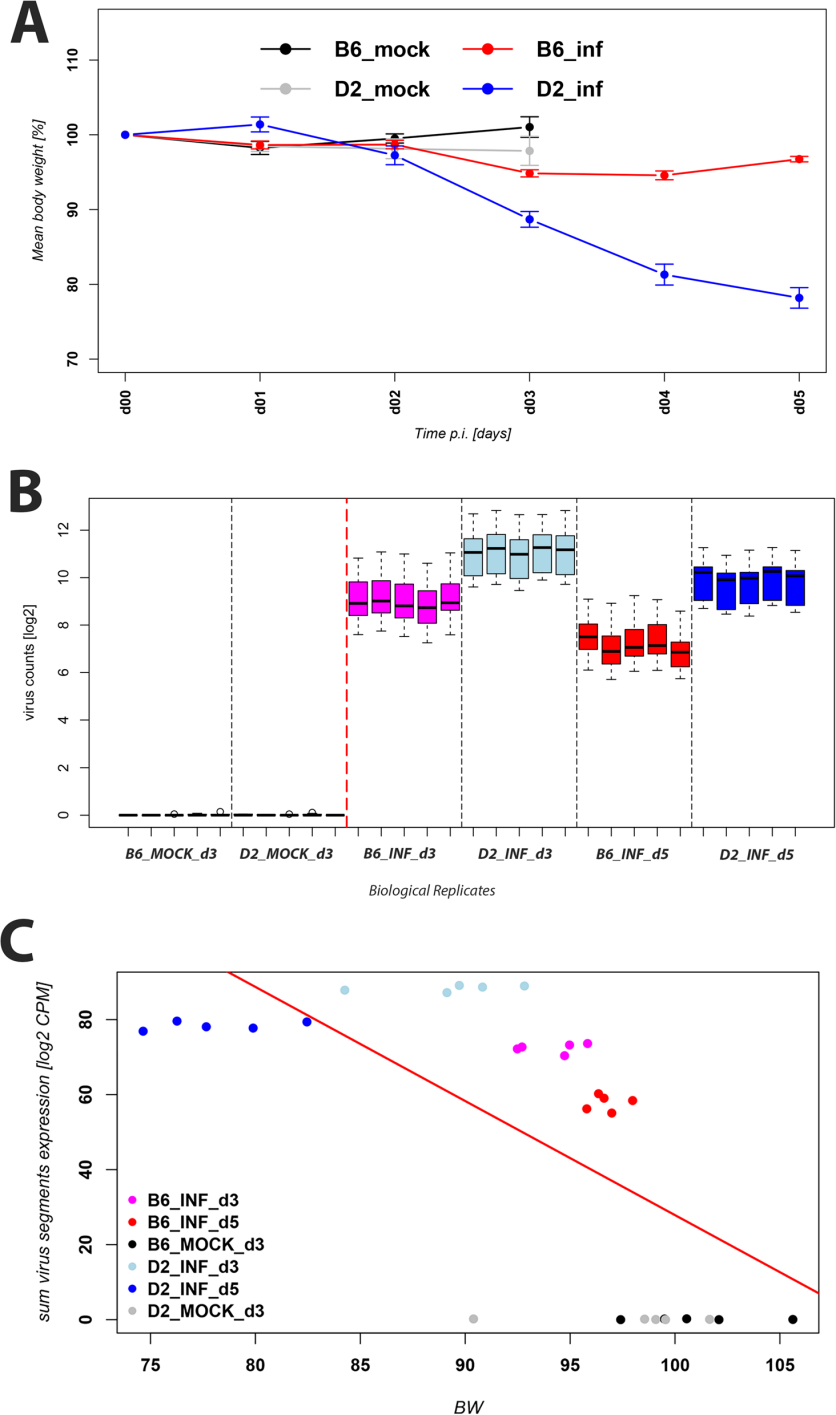
Body weight loss and virus replication. Mice were infected with 2 × 10^3^ FFU of mouse-adapted PRF virus. **A** Body weight was recorded as a percent of the starting weight until mice were sacrificed. The plot shows the mean and +/- 1 SEM of the relative body weight for five biological replicates (mice) per group. **B** Boxplot of virus gene expression in mock-treated and infected B6-*Mx1*^*r/r*^ and D2-*Mx1*^*r/r*^ mice at days 3 and 5 p.i. Each box shows the results for one mouse sample. Samples are organized by groups, each sample representing a biological replicate (mouse). The boxes show the range of expression values (mean and 25% and 75% quartiles of CPMs) for all virus segments for this mouse. **C** Scatter plot representing the relative body weight (percent of starting body weight) for each mouse related to the virus gene expression (log_2_ of the sum of CPMs from all segments) for this mouse. Each mouse is represented by a point. A strong correlation between relative body weight and virus gene expression was observed. Correlation coefficient: -0.67, *p*-value: 6 × 10^−5^

Female B6-*Mx1*^*r/r*^and D2-*Mx1*^*r/r*^ mice (*n* = 5 per group) were infected with 2 × 10^3^ PFU PR8 influenza A virus or PBS only (mock treatment), and body weight was followed for 5 days p.i. (Fig. 1A). Mock-treated controls of both strains did not lose body weight, B6-*Mx1*^*r/r*^ infected mice showed minimal loss of body weight, starting at day 3 p.i., whereas D2-*Mx1*^*r/r*^ infected mice exhibited a larger loss of body weight at day 3 p.i., and significantly higher body weight loss on days 4 and 5 p.i., when compared to B6-*Mx1*^*r/r*^ infected mice. Please note that mock controls were only followed until day 3 when mice were sacrificed.

Expression levels of viral genes in lungs of infected mice can be considered a surrogate for infectious viral load [20]. We therefore determined the expression levels of viral genes at days 3 and 5 p.i. (Fig. 1B). At day 3 p.i. much higher viral gene expression was observed in infected D2-*Mx1*^*r/r*^ mice compared to B6-*Mx1*^*r/r*^ mice. Expression levels decreased in both strains from day 3 to day 5 p.i. but were still much higher in D2-*Mx1*^*r/r*^ (Fig. 1B). These results suggest that B6-*Mx1*^*r/r*^ B6 mice were able to better control initial viral replication and more rapidly cleared virus from the lung compared to D2-*Mx1*^*r/r*^ mice. Furthermore, a strong correlation between gene expression levels of viral genes and body weight loss was observed (Fig. 1C). Only at day 5 p.i., infected D2-*Mx1*^*r/r*^ mice showed a high loss of body weight but lower levels of virus gene expression than D2-*Mx1*^*r/r*^ mice on day 3 p.i. (Fig. 1C). These results suggest that levels of virus replication in D2-*Mx1*^*r/r*^, especially on day 3 p.i., were strongly related to severity, as indicated by body weight loss. The results of body weight analysis on days 0–5 p.i. reproduced the previously observed resistant and susceptible phenotypes to influenza infection in B6-*Mx1*^*r/r*^ and D2-*Mx1*^*r/r*^ mice, respectively [11].

### Analysis of gene expression changes in infected mice

**Fig. 2:**
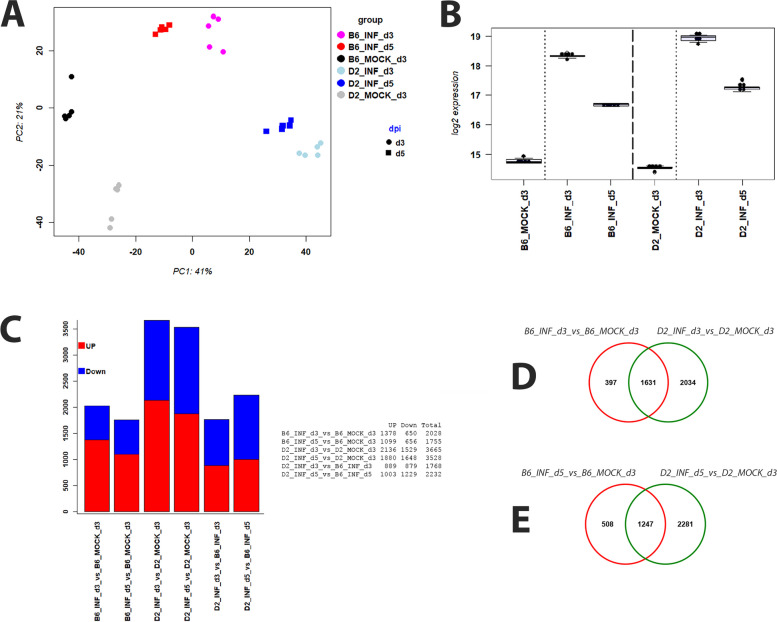
PCA, *Mx1* gene expression and DEGs overview. **A** PC1 and PC2 of a principle component analysis of the normalized transcriptome expression values from infected and mock-treated mouse lungs. Each dot represents values from a single mouse. **B** Boxplot for *Mx1* gene expression values in each group of mice. Each dot represents the value of a single mouse. Boxes represent the mean and range (25% and 75% quartiles) of normalized log_2_ transformed expression values per group. **C** Numbers of up- and down-regulated DEGs for contrasts between the indicated groups of infected B6-*Mx1*^*r/r*^ and D2-*Mx1*^*r/r*^, and mock-treated mice at days 3 and 5 p.i. **D** Venn diagram illustrating overlap of DEGs from contrasts of infected B6-*Mx1*^*r/r*^ and D2-*Mx1*^*r/r*^ versus mock controls at day 3 p.i. **E** Venn diagram illustrating overlap of DEGs from contrasts of infected B6-*Mx1*^*r/r*^ and D2-*Mx1*^*r/r*^ versus mock controls at day 5 p.i

RNA was isolated from infected mice and mock controls and subjected to next-generation sequencing. A principal component analysis (PCA) of normalized gene expression values was performed to determine the main effects of variation. The first two principal components explained 62% of the total variation (Fig. 2A). PC1 and PC2 showed perfect grouping of replicates within a treatment group. PC1 was related to the effect of infection (versus mock), and PC2 to the effect of strains (B6-*Mx1*^*r/r*^ versus D2-*Mx1*^*r/r*^). Expression of *Mx1* was strongly up-regulated in both strains at day 3 p.i. after infection, with higher levels in D2-*Mx1*^*r/r*^ mice and slightly decreased in both strains at day 5 p.i. (Fig. 2B). Thus, the functional *Mx1* allele was also well induced after infection in the susceptible D2-*Mx1*^*r/r*^ mouse strain.

### Differentially expressed genes in infected versus control animals

**Fig. 3:**
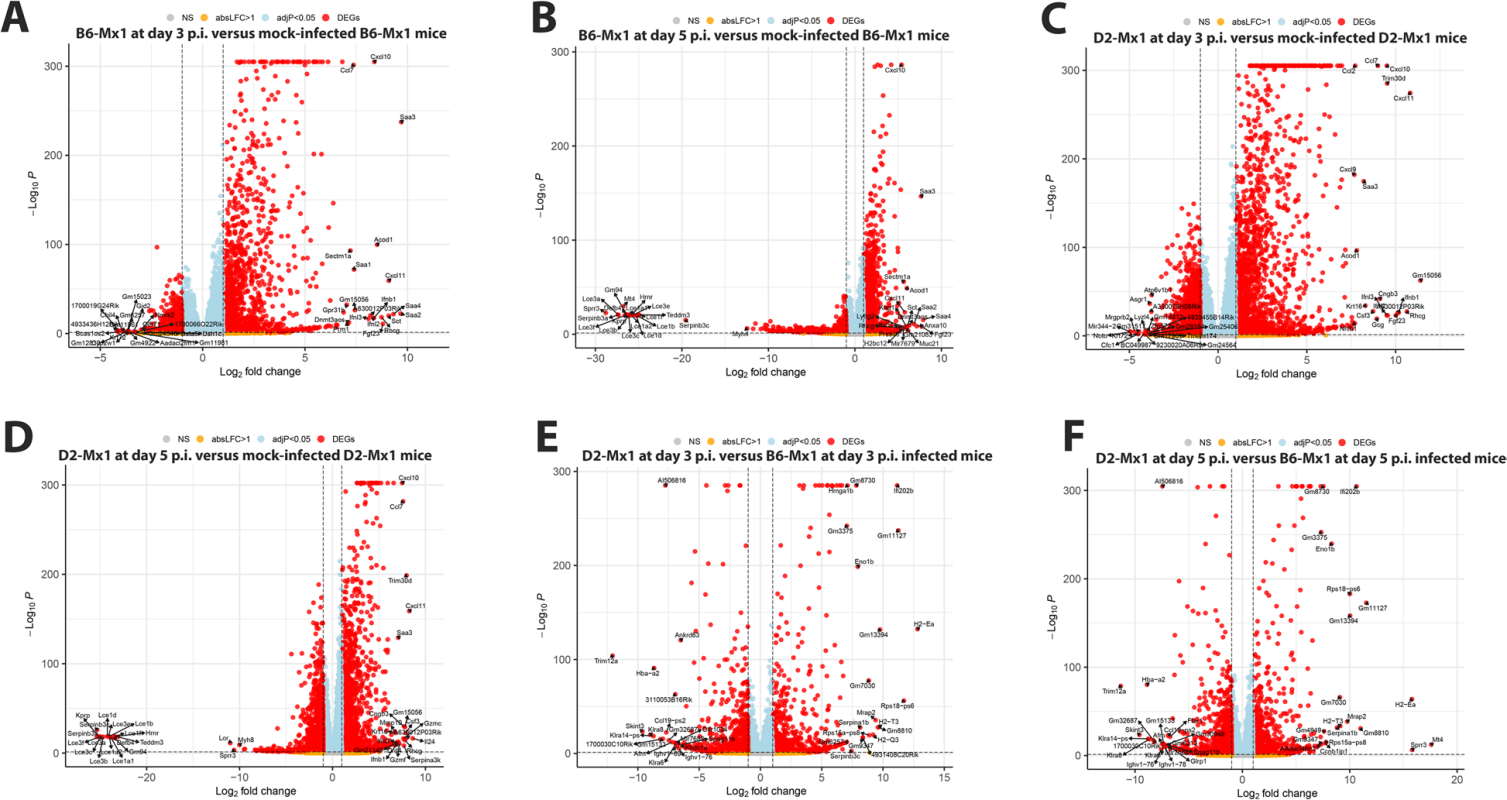
Volcano plots of differentially expressed genes (DEGs). **A** B6-*Mx1*^*r/r*^ at day 3 p.i. versus mock-infected B6-*Mx1*^*r/r*^ mice. **B** B6-*Mx1*^*r/r*^ at day 5 p.i. versus mock-infected B6-*Mx1*^*r/r*^ mice. **C** D2-*Mx1*^*r/r*^ at day 3 p.i. versus mock-infected D2-*Mx1*^*r/r*^ mice. **D** D2-*Mx1*^*r/r*^ at day 5 p.i. versus mock-infected D2-*Mx1*^*r/r*^ mice. **E** D2-*Mx1*^*r/r*^ at day 3 p.i. versus B6-*Mx1*^*r/r*^ at day 3 p.i. infected mice. **F** D2-*Mx1*^*r/r*^ at day 5 p.i. versus B6-*Mx1*^*r/r*^ at day 5 p.i. infected mice. Y-axis: -log_10_ multiple testing adjusted *p*-values, x-axis: log_2_ fold change. DEGs are colored red, and the top 20 up- and down-regulated (by log-fold change) DEGs are labeled. Blue: genes with an adjusted *p*-value < 0.05. Yellow: genes with an absolute log_2_-fold change > 1. Grey: not significant (NS)

We then identified differentially expressed genes between infected B6-*Mx1*^*r/r*^ mice and B6-*Mx1*^*r/r*^ mock controls, between infected D2-*Mx1*^*r/r*^ mice and D2-*Mx1*^*r/r*^ mock controls, and between B6-*Mx1*^*r/r*^ and D2-*Mx1*^*r/r*^ mice on days 3 and 5 p.i. Figure 2C shows the number of up- and down-regulated DEGs for all comparisons. Of note, D2-*Mx1*^*r/r*^ infected mice exhibited much higher numbers of DEGs, compared to B6-*Mx1*^*r/r*^ infected mice at all days p.i., indicating a stronger inflammatory response in D2-*Mx1*^*r/r*^ mice. However, there was a large overlap of DEGs between infected B6-*Mx1*^*r/r*^ and D2-*Mx1*^*r/r*^ versus the respective mock controls; 80% of DEGs in B6-*Mx1*^*r/r*^ were also regulated in D2-*Mx1*^*r/r*^ at day 3 p.i. (Fig. 2D). This overlap was still very high (70%) at day 5 p.i. (Fig. 2E). These observations indicate that the overall regulation of genes after infection was very similar in both mouse strains. Figure 3A to F show the volcano plots of DEGs for all comparisons, demonstrating high quality of the DEG detection with a good range of log-fold changes and *p*-values for all comparisons, and a strong host response in the infected samples at all days p.i. The complete lists of DEGs for all comparisons are provided in Supplementary Tables S1, S2, S3, S4, S5 and S6.

### Functional pathway analysis of differentially expressed genes

**Fig. 4:**
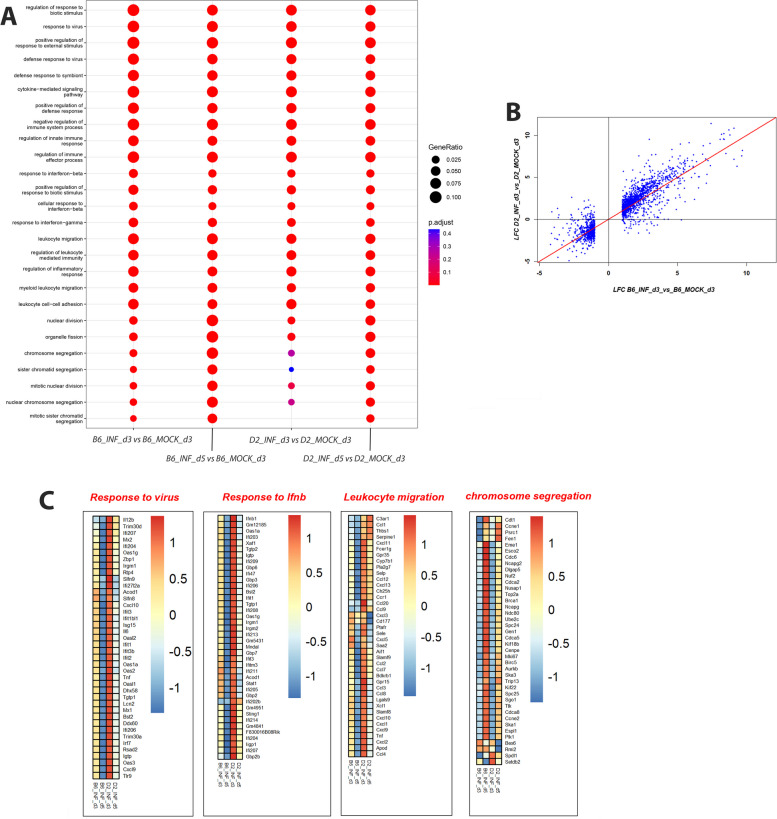
Cluster profiler and heatmaps from pathway analysis of DEGs. **A** Cluster profiler of EnrichGO pathway analysis for up-regulated DEGs from the contrasts of infected mice versus mock-treated controls. **B** Scatter plot showing mean differences as log-fold change (LFC) of infected B6-*Mx1*^*r/r*^ and D2-*Mx1*^*r/r*^ to respective mock treatments for the DEGs from the contrast of B6-*Mx1*^*r/r*^ versus mock at day 3 p.i. **C** Heatmaps of up-regulated DEGs from cluster analysis in **A**, showing the difference in relative gene expression levels in infected mice versus mock-control mice from four pathways, separately for B6-*Mx1*^*r/r*^ and D2-*Mx1*^*r/r*^ mice at days 3 and 5 p.i. Values were scaled by row

Next, we performed a functional pathway analysis of DEGs for the above described comparisons. Figure 4A shows a comparison of the top 30 pathways for the up-regulated DEGs from the contrast of infected to mock controls for both strains at days 3 and 5 p.i. Even though D2-*Mx1*^*r/r*^ infected mice had a much higher number of DEGs, most pathways for B6-*Mx1*^*r/r*^ and D2-*Mx1*^*r/r*^ infected mice were identical. These pathways included Response to virus infections, Response to interferon, Regulation of innate immune response, Leukocyte migration, Chemokine-mediated signaling (Fig. 4A). The pathways were all associated with the host’s response to infection. In addition, Nuclear division, Chromosome segregation pathway activation were observed at day 5 p.i. These pathways most likely reflect the proliferation of infiltrating immune cells and the repair of the lung epithelium, which was damaged by virus infection. However, expression of most DEGs was in general stronger for both up- and down-regulated DEGs in D2-*Mx1*^*r/r*^ mice compared to B6-*Mx1*^*r/r*^ mice at days 3 p.i. (Fig. 4B; expression values of most DEGs in D2-*Mx1*^*r/r*^ were above the diagonal for up-regulated genes and below the diagonal for down-regulated genes).

The heatmaps of expression differences for individual pathways revealed that most genes were higher expressed in the susceptible D2-*Mx1*^*r/r*^ mice compared to the resistant B6-*Mx1*^*r/r*^ mice. Figure 4C shows the expression levels of DEGs from the four main pathways in Fig. 4A. As examples, we list the top five in each pathway and report their known biological functions. In the Response to virus pathway, the five top-ranked DEGs with higher expression in D2-*Mx1*^*r/r*^ on day 3 p.i. compared to B6-*Mx1*^*r/r*^ on day 3 and day 5 were: *Il12b, Trim30d, Ifi207, Mx2*, and *Ifi204* (Fig. 4C; Table 1). In the Interferon beta response pathway, the top five annotated DEGs were: *Ifnb1, Oas1a, Ifi203, Xaf1*, and *Tgtp2* (Fig. 4C; Table 1). In the Leukocyte migration pathway, the five top-ranked DEGs were: *C3ar1, Ccl1, Thbs1, Serpine1*, and *Cxcl11* (Fig. 4C; Table 1). In contrast, for Chromosome segregation pathways, most genes were higher expressed in B6-*Mx1*^*r/r*^ mice compared to D2-*Mx1*^*r/r*^ mice at day 3 p.i.; the top five annotated DEGs were: *Cdt1, Ccne1, Psrc1, Fen1*, and *Eme1* (Fig. 4C; Table 1*)*. In addition, the timing was different from the other pathways, being stronger activated at day 5 p.i. compared to day 3 p.i. Also, down-regulation of inflammatory genes for B6-*Mx1*^*r/r*^ from 3 dpi to 5 dpi was clearly evident (Fig. 4C), most likely due to the elimination of replicating viruses (Fig. 1B).

**Table 1:** List of DEGs identified from analysis of DEGs and their known functions. Information on gene symbols and names from [30], information on gene functions downloaded from [31] and then edited

Gene symbol	Gene name	Known function
*Il12b*	Interleukin 12B	The encoded protein contributes to cytokine activity and cytokine receptor binding activity. It is involved in the regulation of T cell mediated cytotoxicity, T-helper 1 type immune response, and acts in the defense response to other organisms, including cellular responses to lipopolysaccharide, and regulation of type II interferon production.
*Trim30d*	Tripartite motif-containing 30D	The encoded protein has transcription co-activator activity and ubiquitin protein ligase activity. It acts as a defense response to other organisms.
*Ifi207*	Interferon activated gene 207	The encoded protein has double-stranded DNA binding activity and is predicted to be involved in the activation of the innate immune response and the cellular response to interferon-beta.
*Mx2*	MX dynamin-like GTPase 2	The encoded protein has GTP-binding activity and GTPase activity. It is involved in the negative regulation of viral genome replication and the response to type I interferon.
*Ifi204*	Interferon activated gene 204	The encoded protein has double-stranded DNA binding activity and transcription coregulator activity and is involved in cellular response to interferon-alpha. It acts in cellular responses to interferon-beta, positive regulation of osteoblast differentiation, and regulation of transcription by RNA polymerase II.
*Ifnb1*	Interferon beta 1	The encoded protein has cytokine activity and type I interferon receptor binding activity. It is involved in the response to viruses and the type I interferon-mediated signaling pathway.
*Oas1a*	2’-5’ oligoadenylate synthetase 1 A	The encoded protein has 2’-5’-oligoadenylate synthetase activity and double-stranded RNA binding activity. It is involved in the purine nucleotide biosynthetic process and acts upstream of the negative regulation of the viral process.
*Ifi203*	Interferon activated gene 203	The encoded protein has double-stranded DNA binding activity and protein binding activity. It acts upstream of or within the cellular response to interferon-beta.
*Xaf1*	XIAP associated factor 1	The encoded protein has molecular sequestering activity. It is predicted to be involved in negative regulation of protein-containing complex assembly, negative regulation of type I interferon production, and response to interferon-beta. It is thought to act upstream of apoptotic processes.
*Tgtp2*	T cell specific GTPase 2	The encoded protein has GTPase activity. It is predicted to be involved in the defense response to protozoan.
*C3ar1*	Complement component 3a receptor 1	The encoded protein has G protein-coupled receptor activity, complement component C3a binding activity, and complement receptor activity. It is involved in the regulation of angiogenesis, vascular endothelial growth factor production, and granulocyte chemotaxis.
*Ccl1*	C-C motif chemokine ligand 1	The encoded protein has cytokine activity. It is involved in the cellular response to interleukin-17 and acts on cell chemotaxis.
*Thbs1*	Thrombospondin 1	The encoded protein has extracellular matrix-binding activity. It is involved in behavioral responses to pain, regulation of macrophage chemotaxis, and regulation of the transforming growth factor beta receptor signaling pathway. It acts in the cellular response to nitric oxide, circulatory system development, and monocyte aggregation.
*Serpine1*	Serine (or cysteine) peptidase inhibitor, clade E, member 1	The encoded protein has serine-type endopeptidase inhibitor activity. It is involved in the defense response to Gram-negative bacteria, the negative regulation of plasminogen activation, and the regulation of angiogenesis. It acts in cellular responses to the transforming growth factor beta stimulus, placenta development, and regulation of angiogenesis.
*Cxcl11*	Chemokine (C-X-C motif) ligand 11	The encoded protein has CXCR3 chemokine receptor binding activity, chemokine activity, and heparin binding activity. It is involved in the cellular response to lipopolysaccharide, leukocyte chemotaxis, and positive regulation of the release of sequestered calcium ion into the cytosol. It acts on cell chemotaxis and signal transduction.
*Cdt1*	Chromatin licensing and DNA replication factor 1	The encoded protein enables DNA binding. Ii is involved in DNA replication checkpoint signaling and regulation of DNA-templated DNA replication initiation, acting upstream of regulation of nuclear cell cycle DNA replication.
*Ccne1*	Cyclin E1	The encoded protein has cyclin-dependent protein serine/threonine kinase regulator, kinase, and protein kinase binding activity. It acts in DNA metabolic process, homologous chromosome pairing at meiosis, and negative regulation of transcription by RNA polymerase II.
*Psrc1*	Proline/serine-rich coiled-coil 1	The encoded protein has microtubule binding activity. It is involved in microtubule bundle formation, negative regulation of cell growth, and positive regulation of microtubule polymerization.
*Fen1*	Flap structure specific endonuclease 1	The encoded protein has DNA binding, metal ion binding, and nuclease activity. It is involved in DNA repair and DNA replication.
*Eme1*	Essential meiotic structure-specific endonuclease 1	The encoded protein has DNA binding activity. It is predicted to contribute to crossover junction DNA endonuclease activity, to be involved in DNA metabolic process and mitotic intra-S DNA damage checkpoint signaling. It is part of heterochromatin.
*Fcna*	Ficolin A	The encoded protein enables antigen binding. It has carbohydrate derivative binding and signaling receptor binding activity. It is predicted to be involved in complement activation, lectin pathway.
*Hc*	Hemolytic complement	The encoded protein has endopeptidase inhibitor activity. It is involved in positive regulation of angiogenesis and acts upstream in glomerulus development, inflammatory response to wounding, and neutrophil homeostasis.
*Megf10*	Multiple EGF-like-domains 10	The encoded protein has Notch binding activity and is involved in myoblast development, positive regulation of myoblast proliferation, and skeletal muscle satellite cell proliferation. It acts in apoptotic process involved in development, engulfment of apoptotic cell, and recognition of apoptotic cell.
*Cntn2*	Contactin 2	The encoded protein enables cell-cell adhesion mediator activity. It is involved in clustering of voltage-gated potassium channels, establishment of protein localization to juxtaparanode region of axon, and reduction of food intake in response to dietary excess.
*Spon2*	Spondin 2, extracellular matrix protein	The encoded protein enables antigen binding and lipopolysaccharide binding activity. It acts in the defense response to other organism, opsonization, and positive regulation of cytokine production.
*Sftpd*	Surfactant associated protein D	The encoded protein has protein binding activity and is predicted to be involved in negative regulation of interleukin-2 production, opsonization, and regulation of phagocytosis. It acts in innate immune response and respiratory gaseous exchange by respiratory system.
*Cntn6*	Contactin 6	The encoded protein enables cell-cell adhesion mediator activity and acts in positive regulation of Notch signaling pathway.
*Nts*	Neurotensin	The encoded protein has neuropeptide receptor binding activity and receptor ligand activity. It is predicted to be involved antimicrobial humoral immune response mediated by antimicrobial peptide, neuropeptide signaling pathway, and visual learning.
*Ccl17*	C-C motif chemokine ligand 17	The encoded protein has CCR4 chemokine receptor binding activity and chemokine activity and acts in negative regulation of myoblast differentiation.
*Pgc*	Progastricsin (pepsinogen C)	The encoded protein has aspartic-type endopeptidase activity. It is predicted to be involved in positive regulation of antibacterial peptide production and proteolysis.

**Fig. 5:**
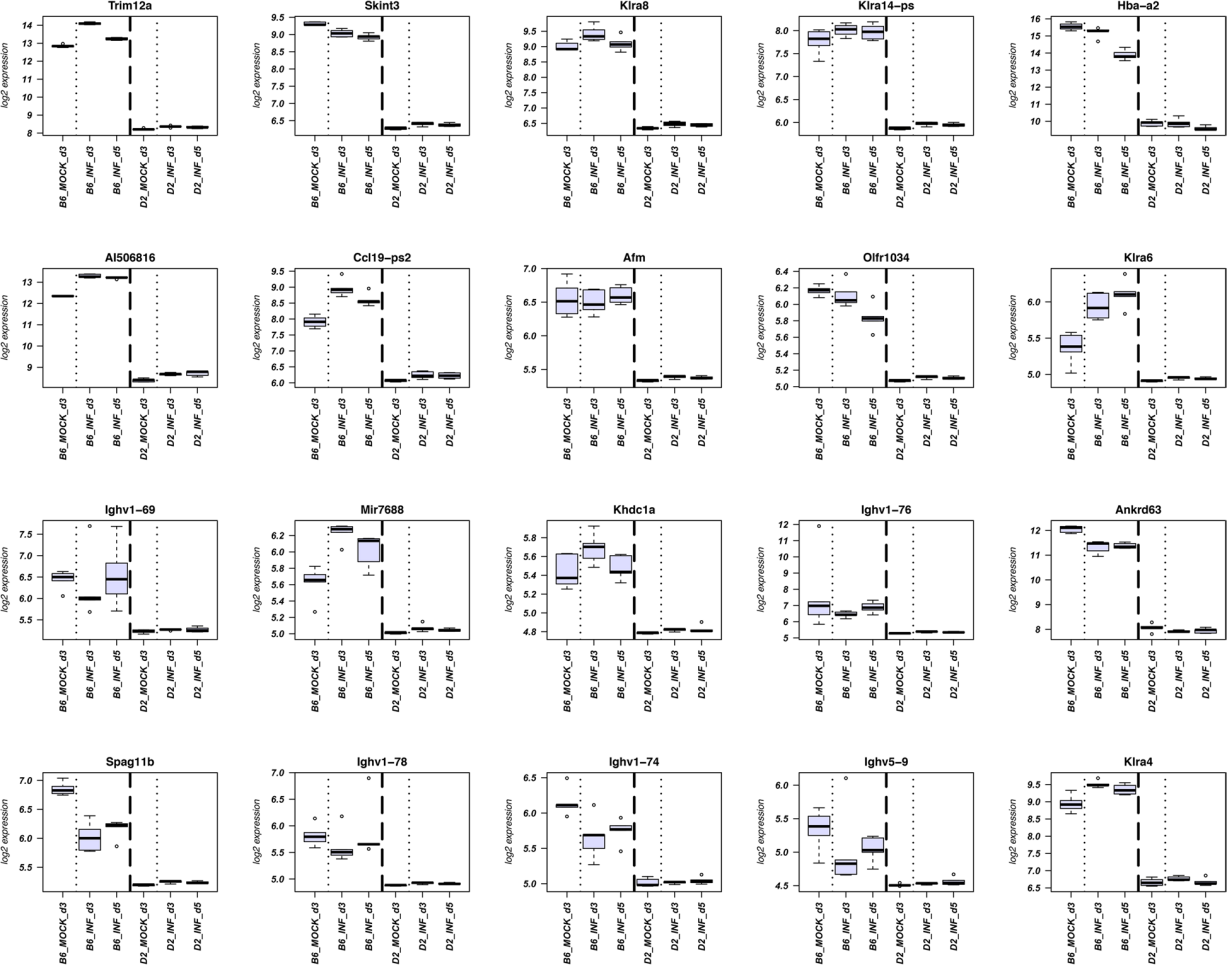
Boxplots for top DEGs up-regulated in B6-*Mx1*^*r/r*^. Boxplots are shown for the top 20 DEGs that were up-regulated (by log-fold-change) in infected B6-*Mx1*^*r/r*^ compared to infected D2-*Mx1*^*r/r*^ at day 3 p.i. and regulated in B6-*Mx1*^*r/r*^ compared to B6-*Mx1*^*r/r*^ mock controls. Boxes represent the mean and range (25% and 75% quartiles) of normalized log_2_ transformed expression values per group

**Fig. 6:**
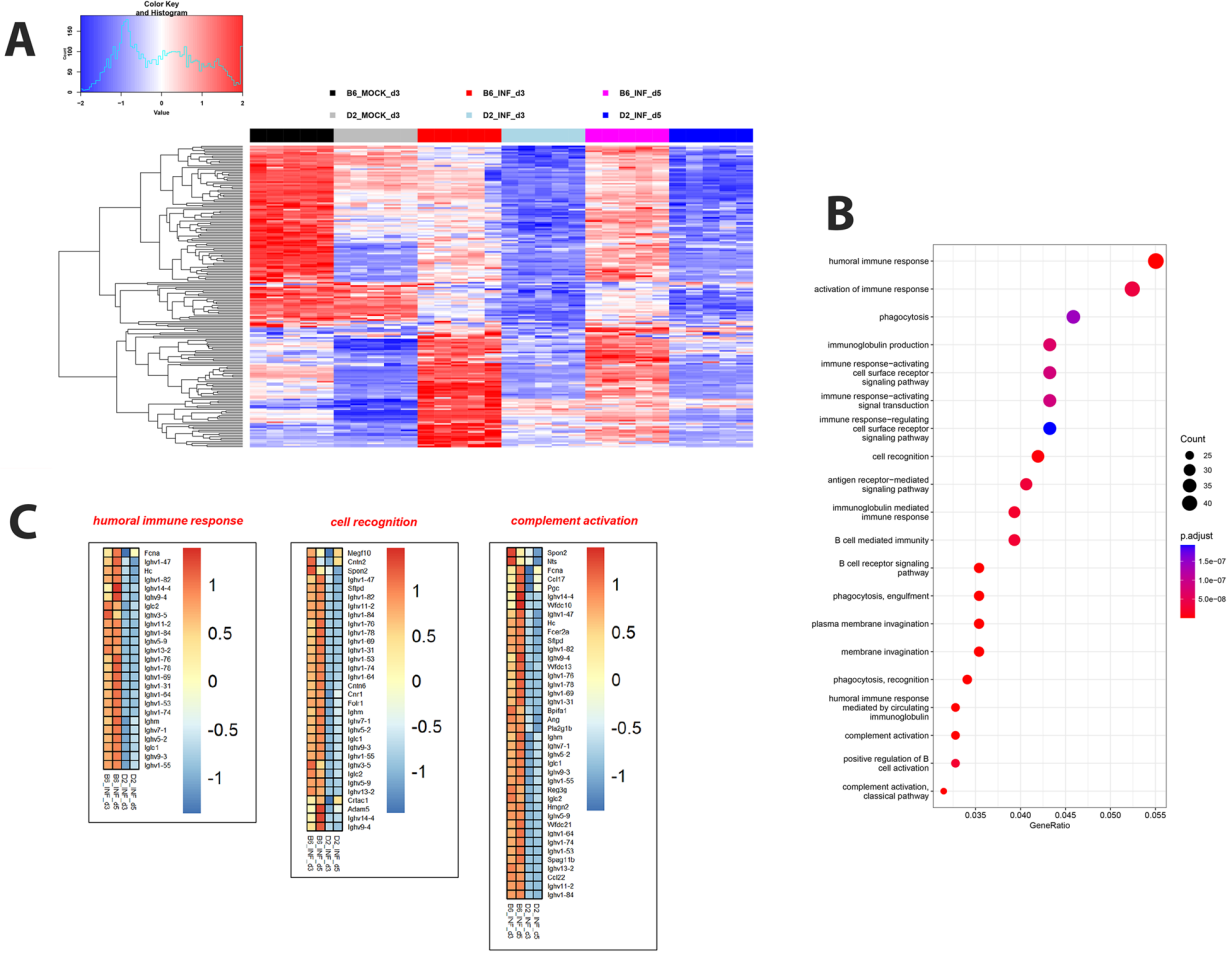
Heatmap, EnrichGO analysis and pathway heatmaps for DEGs up-regulated in B6-*Mx1*^*r/r*^. Results are shown for DEGs that were up-regulated in infected B6-*Mx1*^*r/r*^ compared to infected D2-*Mx1*^*r/r*^ at day 3 p.i. and regulated in B6-*Mx1*^*r/r*^ compared to B6-*Mx1*^*r/r*^ mock controls. **A** Heatmap of normalized expression values for the up-regulated DEGs showing the relative gene expression levels in all groups. Values were scaled by row. **B** Cluster profiler of EnrichGO pathway analysis for DEGs up-regulated in B6-*Mx1*^*r/r*^ mice. **C** Heatmap for relative gene expression levels for infected B6-*Mx1*^*r/r*^ and D2-*Mx1*^*r/r*^ mice, minus mock-controls, for three pathways in Fig. 6B, separately at days 3 and 5 p.i. Values were scaled by row

On the other hand, most important for the activation of the host defense and protection against severe disease are genes that are up-regulated in B6-*Mx1*^*r/r*^, and which may more strongly repress viral replication. We, therefore, looked specifically at genes up-regulated in B6-*Mx1*^*r/r*^ compared to D2-*Mx1*^*r/r*^ at 3 day p.i. and being regulated after infection in B6-*Mx1*^*r/r*^. In total, we identified 717 DEGs belonging to this category (without counting Rik and Gm annotated genes; listed in Table S7). The individual expression levels per group of the top up-regulated 20 DEGs, by LFC, are shown in Fig. 5, an overview of their relative expression levels in a heatmap is shown in Fig. 6A. Both figures clearly demonstrate lower expression in D2-*Mx1*^*r/r*^ at day 3 p.i., which was maintained at day 5 p.i. The main pathways for these DEGs were related to Humoral immune response, Cell recognition, and Complement activation (Fig. 6B). A detailed analysis of DEGs from these pathways identified many Immunoglobulin genes (Fig. 6C). These Ig genes are most likely specific to the B6 haplotype, and thus reads from D2 mice may not be detected in the mapping to the B6 reference genome. As examples for the above DEG pathways genes, we name and discuss the function of the top five genes in each category that were not *Ig* genes (listed in Table 1). In the Humoral immune response pathway, the top-ranked DEGs with higher expression in B6-*Mx1*^*r/r*^ on day 3 p.i. compared to D2-*Mx1*^*r/r*^ were: *Fcna*, and *Hc* (Fig. 6C; Table 1). In the Cell recognition pathway, the top-ranked DEGs with higher expression in B6-*Mx1*^*r/r*^ on day 3 p.i. were: *Megf10, Cntn2, Spon2, Sftpd*, and *Cntn6* (Fig. 6C; Table 1). In the Complement activation pathway, the top-ranked DEGs with higher expression in B6-*Mx1*^*r/r*^ on day 3 p.i. were: *Spon2, Nts, Fcna, Ccl17*, and *Pgc* (Fig. 6C; Table 1).

### Functional pathway analysis of hallmark genes

**Fig. 7:**
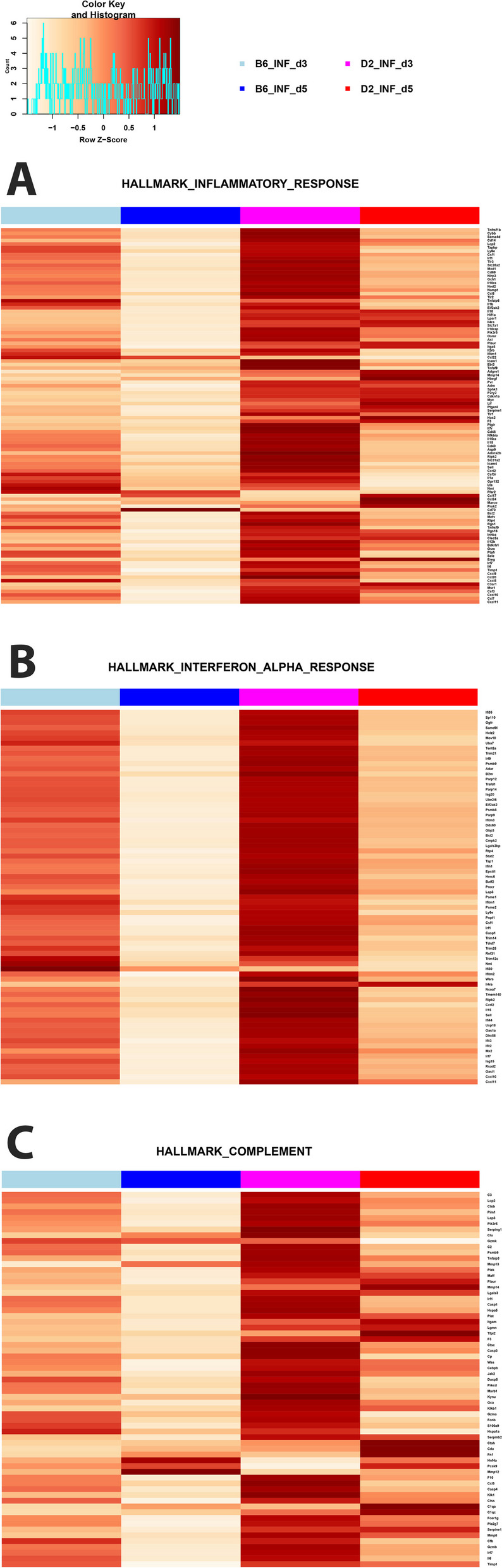
Hallmark gene analysis for DEGs. DEGs from all contrasts of infected D2-*Mx1*^*r/r*^ at day 3 and 5 p.i. versus mock-treated D2-*Mx1*^*r/r*^ and from all contrasts of infected B6-*Mx1*^*r/r*^ at day 3 and 5 p.i. versus mock-treated B6-*Mx1*^*r/r*^ mice were combined (Supplement Table S8) and subjected to hallmark gene analysis. Heatmaps show normalized expression levels (scaled by row) of the means of gene expression levels for B6-*Mx1*^*r/r*^ and D2-*Mx1*^*r/r*^ mice minus the respective controls. **A** Heatmap for hallmark ‘Inflammatory Response’ genes. **B** Heatmap for the hallmark ‘Interferon Alpha’ genes. **C** Heatmap for hallmark ‘Complement’ genes. Values were scaled by row

We then analyzed specific hallmark gene sets [28] of DEGs that were differentially expressed in any comparison of B6-*Mx1*^*r/r*^ and D2-*Mx1*^*r/r*^ mice to the respective mock controls (Table S8). Hallmark genes for Inflammatory response, Interferon-α response, and Complement were almost all higher expressed in infected D2-*Mx1*^*r/r*^ compared to infected B6-*Mx1*^*r/r*^ mice at 3 and 5 days p.i. (Figs. 7A-C). In infected B6-*Mx1*^*r/r*^ mice, the expression of almost all hallmark genes decreased at day 5 p.i. (Figs. 7A-C). In infected D2-*Mx1*^*r/r*^ mice, lower expression was also observed at day 5 p.i. compared to day 3 p.i., but expression was still higher than in B6-*Mx1*^*r/r*^ mice.

### Comparison of gene expression profiles in infected *Mx1*^*r/r*^ to *Mx1*^*-/-*^ mice

Previously, we analyzed differences in lung gene expression in IAV-infected B6-*Mx1*^*−/−*^ and D2-*Mx1*^*−/−*^ mice that were *Mx1*-deficient [20]. Therefore, we compared these responses to responses in mice with a functional *Mx1* gene from this study. Here, we used the ‘standard’ PR8 virus (also referred to PR8F in our previous studies), which is commonly used by other laboratories [11]. This virus is lethal for B6-*Mx1*^*−/−*^ mice at an infection dose of 10^3^ FFU and for D2-*Mx1*^*−/−*^ mice at an infection dose of 10 FFU or lower [11]. For B6-*Mx1*^*r/r*^ mice, infection with this virus is not lethal at an infection dose of 10^3^ FFU [11]. Our previous analyses of *Mx1*-deficient infected mouse strains B6-*Mx1*^*−/−*^ and D2-*Mx1*^*−/−*^ used a much less virulent virus, PR8M [19, 32]. B6-*Mx1*^*−/−*^ mice survive an infection with a dose of 10^3^ FFU PR8M whereas D2-*Mx1*^*−/−*^ mice succumb to the infection [19, 32]. We and others described both viruses in much detail in earlier studies [19, 33]. Furthermore, the analyses with *Mx1*-deficient mice were performed using a different sequencing platform, Ion Torrent [20]. These conditions make it impossible to compare DEGs directly. However, some general trends could be observed, as described below.

**Fig. 8:**
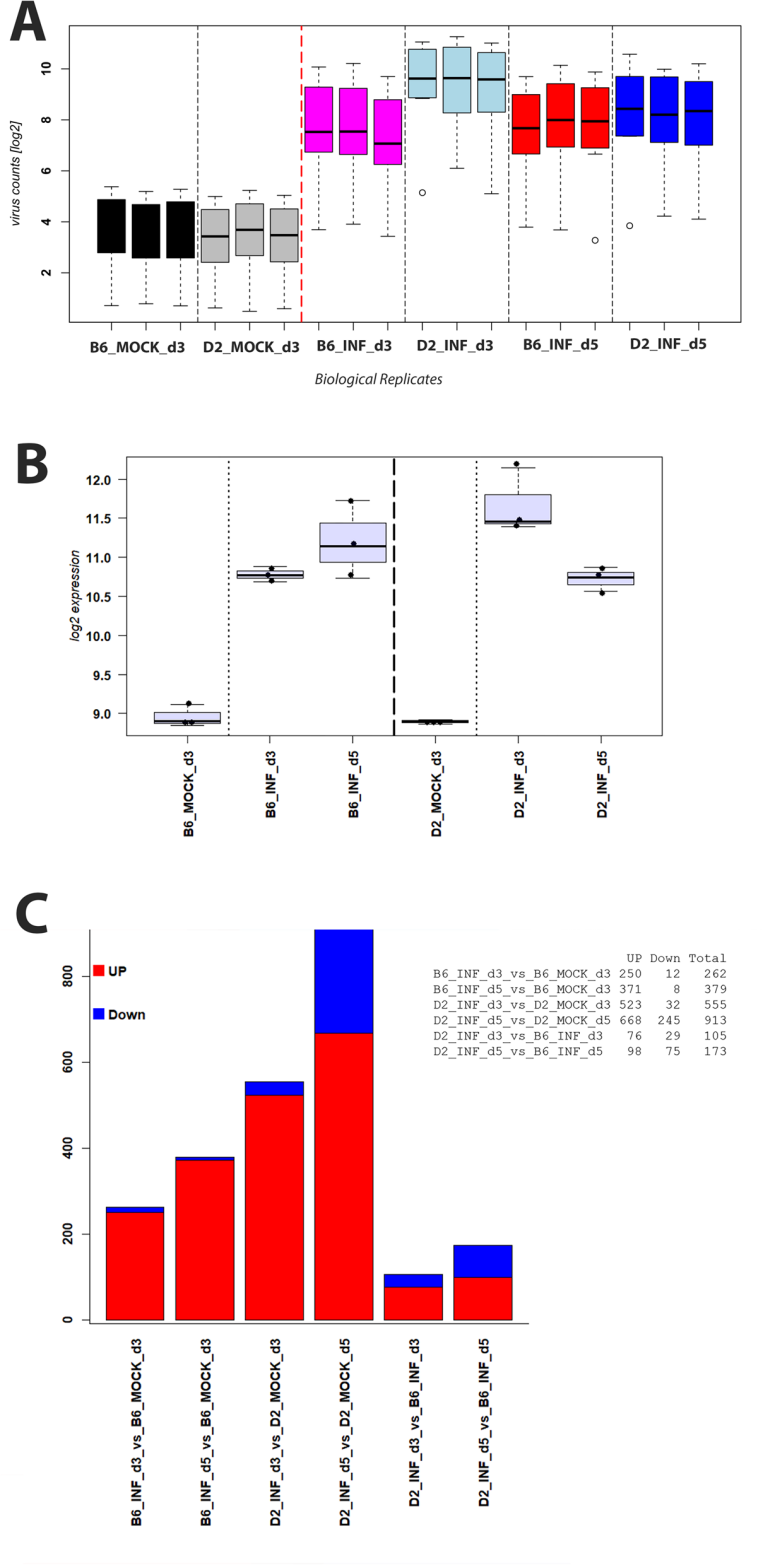
Virus replication and hallmark genes in *Mx1*-deficient mice. **A** Boxplot of virus gene expression in mock-treated and infected B6-*Mx1*^*r/r*^ and D2-*Mx1*^*r/r*^ mice at days 3 and 5 p.i. Each box shows the results for one mouse sample. Samples are organized by groups, each sample representing a biological replicate (mouse). The boxes show the range of expression values (mean and 25% and 75% quartiles of CPMs) for all virus segments for this mouse. Note that because of some host reads mapping to virus genes and subsequent normalization, virus signals in mock-treated mice are not zero but at background levels. **B** Boxplot for *Mx1* gene expression values in each group of *Mx1*-deficient mice. Each dot represents the value from a single mouse. Boxes represent the mean and range (25% and 75% quartiles) of normalized log_2_ transformed expression values per group. **C** Numbers of up- and down-regulated DEGs for contrasts between the indicated groups of infected B6-*Mx1*^*−/−*^ and D2-*Mx1*^*−/−*^ and mock-treated mice at days 3 and 5 p.i

**Fig. 9:**
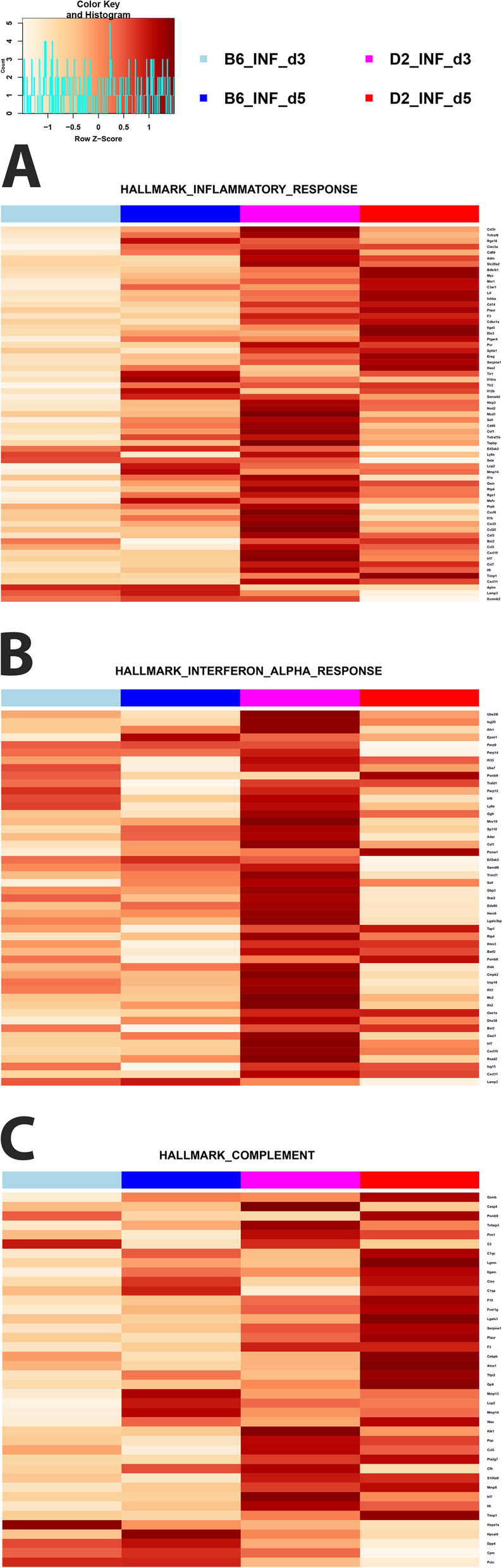
Hallmark gene analysis for DEGs. Heatmaps show normalized expression levels of the means of gene expression levels (scaled by row) for B6-*Mx1*^*−/−*^ and D2-*Mx1*^*−/−*^ mice minus the respective controls for all DEGs (Supplement table S7). **A** Heatmap for hallmark ‘Inflammatory Response’ genes. **B** Heatmap for the hallmark ‘Interferon Alpha’ genes. **C** Heatmap for hallmark ‘Complement’ genes. Values were scaled by row

Most remarkably, the kinetics of virus replication and the number of DEGs was very different in B6-*Mx1*^*−/−*^ and D2-*Mx1*^*−/−*^ mice compared to B6-*Mx1*^*r/r*^ and D2-*Mx1*^*r/r*^ mice. In *Mx1*-deficient mice, viral gene expression in B6-*Mx1*^*−/−*^ was still high at day 3 p.i., whereas it decreased in B6-*Mx1*^*r/r*^ mice between day 3 and 5 p.i. (compare Fig. 1B with 8A). Also, *Mx1* transcripts were up-regulated after infection with expression kinetics resembling the ones observed for *Mx1*^*r/r*^ mice (compare Figs. 2B, 3, 4, 5, 6, 7 and 8B). Only in B6-*Mx1*^*r/r*^ mice, expression at day 5 p.i. was lower compared to day 3 p.i. whereas in B6-*Mx1*^*−/−*^ mice, expression was higher at day 5 p.i. than on day 3 p.i. Furthermore, the number of DEGs increased from day 3 to day 5 p.i. (Fig. 8A) in both B6-*Mx1*^*−/−*^ and D2-*Mx1*^*−/−*^, whereas in B6-*Mx1*^*r/r*^ and D2-*Mx1*^*r/r*^ mice, the numbers of DEGs decreased (compare Figs. 2C, 3, 4, 5, 6, 7 and 8C). Expression of hallmark genes was still high in B6-*Mx1*^*−/−*^ mice at day 5 p.i. compared to day 3 p.i. (Figs. 9A-C) whereas in B6-*Mx1*^*r/r*^ mice, they decreased from day 3 to day 5 p.i. (compare Figs. 7A-C, 8 and 9A-C). In D2-*Mx1*^*−/−*^ mice, the Interferon alpha response was still high at day 5 p.i. compared to day 3 p.i. (Fig. 9B) and did not decrease as in D2-*Mx1*^*r/r*^ mice (Fig. 7B). Complement activation hallmark genes were higher at day 5 p.i. than day 3 p.i. in D2-*Mx1*^*−/−*^ mice (Fig. 9C) whereas in D2-*Mx1*^*r/r*^ mice, the expression levels decreased from day 3 to day 5 p.i. (Fig. 7C).

## Discussion

Here, we report on a detailed analysis of gene expression changes in the lungs of B6-*Mx1*^*r/r*^ and D2-*Mx1*^*r/r*^ mice, which carry the same functional *Mx1* gene derived from A2G mice. B6-*Mx1*^*r/r*^ were highly resistant and survived an infection, whereas D2-*Mx1*^*r/r*^ were highly susceptible and died.

Even though D2-*Mx1*^*r/r*^ infected mice activated a much higher number of DEGs, most pathways for B6-*Mx1*^*r/r*^ and D2-*Mx1*^*r/r*^ infected mice were identical. These findings suggest that gene expression profiles in both mouse models were mainly directed by the infection and not directly associated with the presence of a functional *Mx1* gene. However, virus replication was strongly suppressed in B6-*Mx1*^*r/r*^ mice. Thus, the presence or absence of a functional *Mx1* gene does not seem to have an effect on the overall gene expression profiles, but rather directly affects viral replication. Many DEGs were identified in the comparison of infected B6-*Mx1*^*r/r*^ versus their mock controls and infected D2-*Mx1*^*r/r*^ versus their mock controls, as well as in the contrast of infected B6-*Mx1*^*r/r*^ versus infected D2-*Mx1*^*r/r*^ mice. In general, D2-*Mx1*^*r/r*^ infected mice showed much higher numbers of DEGs, compared to B6-*Mx1*^*r/r*^ infected mice. Similar observations were made for selected hallmark genes. These general differences were most likely due to the higher viral loads causing a stronger activation of host response genes in D2-*Mx1*^*r/r*^ mice.

Most important for understanding resistance in B6-*Mx1*^*r/r*^ mice are genes that are up-regulated in B6-*Mx1*^*r/r*^ but not or to a lesser extent in D2-*Mx1*^*r/r*^. Our analysis identified many DEGs in various analyses (a summarized selection is shown in Table 1) that may play an important role in the host defense and/or explain the difference in susceptibility to IAV in B6-*Mx1*^*r/r*^ versus D2-*Mx1*^*r/r*^ mice. We describe the known function of these genes in Table 1 (using information from [30] and [31]). Almost all these genes play an important role in the host response against microbial infections or damage repair after infection.

In particular, genes from the humoral immune response were activated in B6-*Mx1*^*r/r*^ but not in D2-*Mx1*^*r/r*^ mice early after infection. These findings suggest that in D2-*Mx1*^*r/r*^ mice, although a high inflammatory response was generated, some arms of the host immune response were not properly functioning. This defect may already exist before the infection event, and all observed differences and defects that we observe are secondary to the initial defect. Of note, many of these DEGs showed already low or no expression in D2-*Mx1*^*r/r*^ compared to B6-*Mx1*^*r/r*^ at baseline in mock-treated mice (Fig. 5). Alternatively, very early responses are compromised, which allowed high virus replication early after infection. The finding that alveolar macrophages showed a dysfunctional phenotype in D2 mice supports the hypothesis of a pre-infection defect [34].

The function of genes that were expressed higher in B6-*Mx1*^*r/r*^ may explain the stronger resistance of B6-*Mx1*^*r/r*^ mice to infections with influenza A virus. However, a large overlap of activated DEGs was observed between B6-*Mx1*^*r/r*^ and D2-*Mx1*^*r/r*^. This observation suggest that the cause for the high susceptibility of D2 mice was most likely not due to a single gene but represents a complex trait with multi-genic effects. We and others have shown in quantitative trait mapping studies using BXD genetic reference populations that the stronger resistance of B6-*Mx1*^*−/−*^ compared to D2-*Mx1*^*−/−*^ mice was not due to a single gene but most likely linked to the function of many gene loci [35, 36]. In line with this finding, the ATPase SMARCA2, which is a component of the SWI/SNF chromatin remodeling complex and a co-transcriptional regulator of many ISGs, was identified as a cofactor for human MX1-mediated antiviral activity against IAV. However, this was not due to a direct effect on the MX1 protein, but rather by regulating gene expression of important viral restriction factors such as IFITM2 or IGFBP3 that may work in concert with MX1 [37].

Of note, the expression kinetics of *Mx1* transcripts themselves in *Mx1*-deficient mice and *Mx1*^*r/r*^ mice for both strains were very similar, indicating that in *Mx1*-deficient mice, regulation of the gene was not impaired. Only in B6-*Mx1*^*r/r*^ mice, expression at day 5 p.i. was lower compared to day 3 p.i. whereas in B6-*Mx1*^*−/−*^ mice, expression was higher at day 5 p.i. than on day 3 p.i. These observations are in line with the effective reduction of viral load in B6-*Mx1*^*r/r*^ mice from day 3 to 5 p.i.

We also compared the results from this study to gene expression changes in *Mx1*-deficient mice, which were published previously [20]. In *Mx1*-deficient B6-*Mx1*^*−/−*^ and D2-*Mx1*^*−/−*^ mice, the number of DEGs was much higher at day 5 compared to day 3 p.i. whereas in B6-*Mx1*^*r/r*^ and D2-*Mx1*^*r/r*^ mice, the number of DEGs at day 5 was lower than at day 3. These results suggest that in both mouse strains, carrying a functional *Mx1*, virus replication was repressed more efficiently than in *Mx1*-deficient mice. However, this did not rescue D2-*Mx1*^*r/r*^ mice from death, most likely because lung damage caused by early high virus replication and spread, and immunopathology was already too advanced and could not be reversed.

From all the above results on viral loads and expression of DEGs, we conclude that the high susceptibility in D2-*Mx1*^*r/r*^ mice was most likely due to a combination of a high viral load, leading to lung tissue damage, and the hyper-inflammatory immunopathology caused by a strong anti-inflammatory reaction of the innate immune system. This hypothesis is further supported by our previous studies showing higher chemokine/cytokine secretion and viral loads in the infected lungs of D2-*Mx1*^*r/r*^ mice [11].

During very early time points, expression of interferons and their response genes is suppressed in virus-infected cells by viral genes, and virus replication increases exponentially [38]. Infected cells secrete interferons which induced an anti-viral response in non-infected genes. Thus, expression of a functional *Mx1* is activated at a time when the virus has already massively spread in the lungs of D2-*Mx1*^*r/r*^ mice and is not able to substantially limit lung damage caused by the early virus spreading.

D2-*Mx1*^*r/r*^ mice can be rescued by a pre-infection treatment with interferons [11] or by treatment with defective interfering particles [13]. These observations showed that *Mx1* is fully functional in D2-*Mx1*^*r/r*^ mice, and when activated prior to infection, it is able to suppress early virus replication and prevent death. One reason for the higher and more rapid early virus replication in D2 mice may be a dysfunction of alveolar macrophages and an increased permissiveness of respiratory cells to virus infection [34].

Phenotypic and molecular analyses in some mouse strains that are wild-derived, and which carry a function *Mx1* gene have been described. In general, mice carrying a functional *Mx1* gene strongly suppress viral replication, whereas mice with a non-functional *Mx1* gene carry higher viral loads [39, 40]. The exceptions are D2-*Mx1*^*r/r*^ [11] and CAST/EiJ mouse strains [39, 40]. In these strains, the duration and high magnitude of the expression of host inflammatory genes strongly correlates with viral loads [40–43], supporting the hypothesis that the destruction of lung tissue by high virus replication and spread, and the strong hyperinflammatory response lead to morbidity and mortality in these susceptible mouse strains.

Our study has some limitations. We identified many genes that may play an important role in explaining the difference between susceptible and resistant mice. However, future experimental studies will be necessary to actually demonstrate such a function during influenza or viral infections. For example, DEGs between B6-*Mx1*^*r/r*^ and D2-*Mx1*^*r/r*^ could be functionally tested in B6-*Mx1*^*r/r*^ mouse knock-out mutants for reproducing a D2-*Mx1*^*r/r*^ susceptible phenotype. Also, our studies have been performed in the mouse model, and their importance for human infections will have to be demonstrated in human cell culture, organoids, lung tissue cultures or genome wide association studies. Nevertheless, we identified highly valuable candidates that would merit such analyses in the human system. Also, D2-*Mx1*^*r/r*^ mice at day 5 p.i. were highly moribund, most likely due to the destruction of lung tissue, which resulted in massive cell death, compromising further virus replication. Therefore, expression of genes may be reduced in general because of the high destruction of lung tissue. We thus concentrated our study on the earlier stages, at day 3 p.i. by trying to identify important genes in B6-*Mx1*^*r/r*^ that may explain its resistance compared to highly susceptible D2-*Mx1*^*r/r*^ mice. Furthermore, we compared our results in *Mx1* mice with findings from *Mx1*-deficient mice, and we observed some general trends that were different. However, *Mx1*-deficient mice were infected with a much less virulent virus, and expression analysis used a different next-generation sequencing platform [20] which makes a direct comparison difficult.

## Conclusions

In this study, we performed a detailed transcriptome analysis in the lungs of B6-*Mx1*^*r/r*^ and D2-*Mx1*^*r/r*^ mice carrying a functional *Mx1* gene after infection with the influenza A virus. B6-*Mx1*^*r/r*^ were highly resistant to virus infections, they lost little weight and survived, whereas D2-*Mx1*^*r/r*^ mice were highly susceptible, losing weight rapidly and dying. We identified many differentially expressed genes in D2-*Mx1*^*r/r*^ compared to B6-*Mx1*^*r/r*^ mice at days 3 and 5 p.i. However, the overall activation of host response pathways was similar in both strains. Thus, the presence or absence of a functional *Mx1* gene did not seem to have an effect on the overall gene expression profiles. We identified many DEGs that showed higher expression levels in B6-*Mx1*^*r/r*^ compared to D2-*Mx1*^*r/r*^ mice. These genes may be involved in resistance to influenza infections.

We hypothesize that the activation of certain immune response genes was missing and that others, especially *Mx1*, were expressed at a time in D2-*Mx1*^*r/r*^ mice virus had already massively spread in the lung and were thus not able to protect them from severe disease.

### Supplementary Information

**Additional file 1. **Description of data: list of DEGs from comparison of infected B6-*Mx1*^*r/r*^ at day 3 p.i. versus B6-*Mx1*^*r/r*^ mock controls.

**Additional file 2. **Description of data: list of DEGs from comparison of infected B6-*Mx1*^*r/r*^ at day 5 p.i. versus B6-*Mx1*^*r/r*^ mock controls at day 5 p.i.

**Additional file 3. **Description of data: list of DEGs from comparison of infected D2-*Mx1*^*r/r*^ at day 3 p.i. versus D2-*Mx1*^*r/r*^ mock controls.

**Additional file 4. **Description of data: list of DEGs from comparison of infected D2-*Mx1*^*r/r*^ at day 5 p.i. versus D2-*Mx1*^*r/r*^ mock controls.

**Additional file 5. ** Description of data: list of DEGs from comparison of infected D2-*Mx1*^*r/r*^ versus B6-*Mx1*^*r/r*^ at day 3 p.i.

**Additional file 6. **Description of data: list of DEGs from comparison of infected D2-*Mx1*^*r/r*^ versus B6-*Mx1*^*r/r*^ at day 3 p.i.

**Additional file 7. **Description of data: DEGs up-regulated in B6-*Mx1*^*r/r*^ and regulated in infected B6-*Mx1*^*r/r*^ at day 3 p.i. (all DEGs in B6-*Mx1*^*r/r*^ versus mock controls).

**Additional file 8. **Description of data: combined list of all DEGs from comparison of infected D2-*Mx1*^*r/r*^ and B6-*Mx1*^*r/r*^ mice day 3 and 5 p.i. versus controls.

## Data Availability

The raw data and normalized gene expression levels are available at the GEO expression database [44, 45]. ID: GSE252374.
