# Supplementary material for: Differential lung gene expression changes in C57BL/6 and DBA/2 mice carrying an identical functional Mx1 gene reveals crucial differences in the host response
Source: BMC Genom Data. 2024 Feb 15;25:19. doi: 10.1186/s12863-024-01203-3 (PMC10870463; doi:10.1186/s12863-024-01203-3)
Supplement: Supplementary file 8 — Additional file 8. Description of data: combined list of all DEGs from comparison of infected D2-Mx1r/r and B6-Mx1r/r mice day 3 and 5 p.i. versus controls. [file 12863_2024_1203_MOESM8_ESM.pdf]

Supplementary material

Table S1

Title: S1_DEG_DESeq2_B6Mx1_PR8F_d3_vs_B6Mx1_MOCK_d3_111223.xlsx. Description of data: list of DEGs from comparison of infected B6-*Mx1*$^{r/r}$ at day 3 p.i. versus B6-*Mx1*$^{r/r}$ mock controls.

Table S2

Title: S2_DEG_DESeq2_B6Mx1_PR8F_d5_vs_B6Mx1_MOCK_d3_111223.xlsx. Description of data: list of DEGs from comparison of infected B6-*Mx1*$^{r/r}$ at day 5 p.i. versus B6-*Mx1*$^{r/r}$ mock controls at day 5 p.i.

Table S3

Title: S3_DEG_DESeq2_D2Mx1_PR8F_d3_vs_D2Mx1_MOCK_d3_111223.xlsx. Description of data: list of DEGs from comparison of infected D2-*Mx1*$^{r/r}$ at day 3 p.i. versus D2-*Mx1*$^{r/r}$ mock controls.

Table S4

Title: S4_DEG_DESeq2_D2Mx1_PR8F_d5_vs_D2Mx1_MOCK_d3_111223.xlsx. Description of data: list of DEGs from comparison of infected D2-*Mx1*$^{r/r}$ at day 5 p.i. versus D2-*Mx1*$^{r/r}$ mock controls.

Table S5

Title: S5_DEG_DESeq2_D2Mx1_PR8F_d3_vs_B6Mx1_PR8F_d3_111223.xlsx. Description of data: list of DEGs from comparison of infected D2-*Mx1*$^{r/r}$ versus B6-*Mx1*$^{r/r}$ at day 3 p.i.

Table S6

Title: S6_DEG_DESeq2_D2Mx1_PR8F_d5_vs_B6Mx1_PR8F_d5_111223.xlsx. Description of data: list of DEGs from comparison of infected D2-*Mx1*$^{r/r}$ versus B6-*Mx1*$^{r/r}$ at day 3 p.i.

Table S7

Title: S7_D2vsB6_DN_d3_regulated_B6d3_121223.xlsx. Description of data: DEGs up-regulated in B6-*Mx1*$^{r/r}$ and regulated in infected B6-*Mx1*$^{r/r}$ at day 3 p.i. (all DEGs in B6-*Mx1*$^{r/r}$ versus mock controls).

Table S8

Title: S8_all_DEGs_INFvsCTRL_111223.xlsx. Description of data: combined list of all DEGs from comparison of infected D2-*Mx1*$^{r/r}$ and B6-*Mx1*$^{r/r}$ mice day 3 and 5 p.i. versus controls.
